# MiRNA-132/212 regulates tight junction stabilization in blood–brain barrier after stroke

**DOI:** 10.1038/s41420-021-00773-w

**Published:** 2021-12-08

**Authors:** Haomin Yan, Hideaki Kanki, Shigenobu Matsumura, Tomohiro Kawano, Kumiko Nishiyama, Shintaro Sugiyama, Hiroshi Takemori, Hideki Mochizuki, Tsutomu Sasaki

**Affiliations:** 1 Department of Neurology, Graduate School of Medicine, Osaka University, Yamadaoka 2-2, Suita, Osaka, 565-0871 Japan; 2 Laboratory of Nutrition Chemistry, Division of Food Science and Biotechnology, Graduate School of Agriculture, Kyoto University, Kyoto, Japan; 3 Department of Chemistry and Biomolecular Science, Faculty of Engineering, Gifu University, Gifu, Japan

**Keywords:** Neuro-vascular interactions, Blood-brain barrier

## Abstract

MicroRNA-132/212 has been supposed as a critical gene related to the blood–brain barrier (BBB) protection after stroke, but its regulation pathway including the upstream regulator and downstream targets is still unclear. Herein, we demonstrated the cAMP response element-binding protein (CREB)-regulated transcription coactivator-1 (CRTC1) to be the upstream regulator of miRNA-132/212 using CRTC1 knockout and wild-type mice. CRTC1 deletion led to the reduction of miRNA-132/212 expression in mice brain after ischemic stroke, significantly increased infarct volume, and aggravated BBB permeability with worsening neurological deficits. Furthermore, we identified that miRNA-132 repressed Claudin-1, tight junction-associated protein-1 (TJAP-1), and RNA-binding Fox-1 (RBFox-1) by directly binding to their respective 3′-untranslated regions, which alleviated the ischemic damage by enhancing neuronal survival and BBB integrity. Moreover, the co-culture of endothelial cells with CRTC1-deficient neurons aggravated the cell vulnerability to hypoxia, also supporting the idea that miRNA-132/212 cluster is regulated by CRTC1 and acts as a crucial role in the mitigation of ischemic damage. This work is a step forward for understanding the role of miRNA-132/212 in neurovascular interaction and may be helpful for potential gene therapy of ischemic stroke.

## Introduction

Stroke is a leading cause of mortality and long-term disability worldwide [1, 2]. Although intensive efforts have been made to develop new pharmacological therapies, tissue plasminogen activator is still the only Food and Drug Administration-approved medication for ischemic stroke, but its use is restricted to a narrow time window, with a high risk of hemorrhage [3]. Therefore, a new treatment approach with an extended therapeutic time window is urgently required for ischemic stroke.

MicroRNAs (miRNAs), a group of endogenous non-coding RNAs composed of ~20 nucleotides, function as a critical role in post-transcriptional gene regulation via sequence-specifically binding to the 3′-untranslated region (UTR) of one or more messenger RNAs (mRNAs) [4]. Previous studies have demonstrated that miRNA conservation is often cell-type and tissue-specific [5], implying that miRNAs play a functional role in intercellular interactions.

MiR-132/212 is a highly brain-enriched miRNA cluster and has been well studied as an upstream inhibitory regulator on mRNA expression in CNS [6]. Zuo et al. have recently reported that miR-132 attenuates cerebral injury via repressing MMP9 [7]. Otherwise, increasing studies suggested miR-132/212 to be a downstream regulator mediated by cAMP response element-binding protein (CREB) in visual plasticity [8], memory [9, 10], and pain regulation [11]. Our previous studies demonstrated that CREB-dependent gene transcription is mainly dependent on CREB-regulated transcription coactivator-1 (CRTC1) coactivation [12], and CRTC1–CREB complex exerts a neuroprotective effect by regulating several factors, including proliferator-activated receptor gamma coactivator-1 alpha (PGC-1α) [13, 14] and brain-derived neurotrophic factor (BDNF) [15]. Also, miR-132 is demonstrated to be regulated by CRTC1 [16]. Inspired by these studies, we hypothesized that the CRTC1 may be an upstream regulator of miRNA-132/212.

The disruption of blood–brain barrier (BBB) was one of the most common pathological events in the very acute stage of ischemic stroke [17, 18]. As a highly dynamic and complex structure, BBB is composed of pericytes, astrocytes, endothelial cells, and basement membrane, and regulates the movement of substances between CNS and blood [19, 20]. It is known that the BBB function is regulated by certain tight junction protein. Claudin-5, as the most highly expressed tight junction protein at BBB, its dysfunction is closely associated with ischemic stroke pathology [21]. BBB permeability was found to be enhanced in Claudin-5 deficient mice, as under physiological condition, the 443 Da tracer leaked from the blood vessel to the parenchymal side of brain, but the 1.9 kDa tracer did not leak [22]. Recently, Sladojevic et al. reported Claudin-1 accumulated in leaky brain microvessels after stroke, and Claudin-1 specifically blockage had beneficial effects on BBB permeability [23]. These studies demonstrated the crucial role of tight junction proteins like Claudins in BBB integrity maintenance, but their upstream mechanism in stroke remains unknown.

To reveal the CRTC1-miR-132/212 signaling in the acute ischemic stroke phase, in this work we generated and subjected CRTC1 knockout and wild-type mice to 60 min transient middle cerebral artery occlusion (MCAO). Our findings showed that CRTC1 deficiency decreased miR-132/212 expression and aggravated mice neurological outcome after ischemic stroke. Furthermore, we identified and validated Claudin-1, tight junction-associated protein-1 (TJAP-1), and RNA-binding Fox-1 (RBFox-1) as targets of miR-132/212, which play a crucial role in the homeostasis of neuron–endothelial interaction under hypoxia. Besides, the in vitro experiments also suggested that CRTC1-mediated regulation of miR-132/212 on neuron–endothelial interaction act as a crucial role in the mitigation of cell susceptibility to hypoxia. This work offers new clues for understanding the role of miRNA-132/212 in neurovascular regulation and may provide a reference for potential novel therapy by controlling tight junction complex such as Claudin-1 and TJAP-1 after ischemic stroke.

## Results

### CRTC1 deficiency suppresses the upregulation of miR-132/212 after cerebral ischemia in mice

**Fig. 1:**
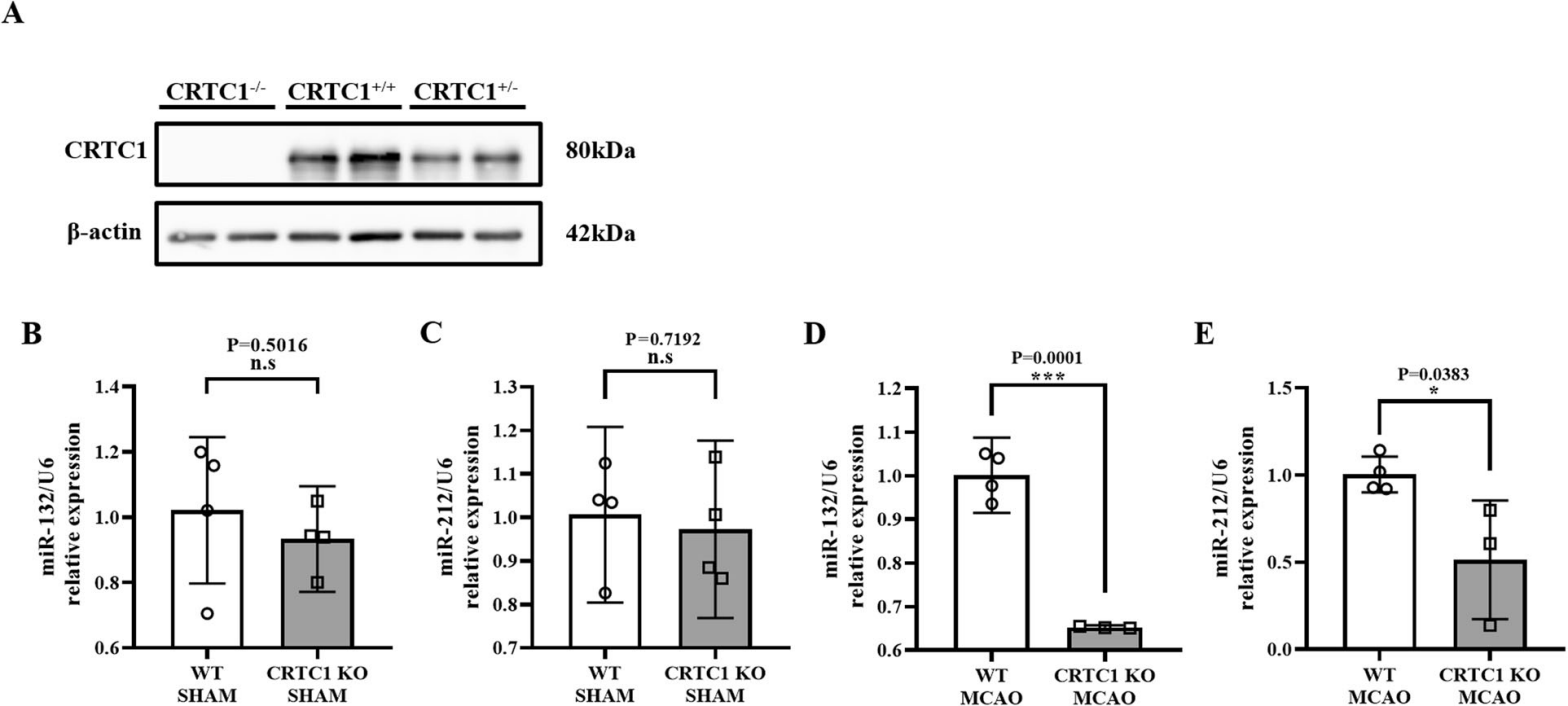
CRTC1 deficiency induces miR-132/212 degradation after MCAO. **A** Western Blot shows CRTC1 has been completely deleted on C57B/6 background mouse. Quantitative polymerase chain reaction (qPCR) results of miR-132 (**B**) and miR-212 (**C**) in CRTC1 KO and WT mice brain under sham group. qPCR results of miR-132 (**D**) and miR-212 (**E**) in penumbra regions of CRTC1 KO and WT mice brain 24 h after MCAO. *n* = 3–4 for each group.

To investigate the changes in miR-132/212 in ischemia, we induced MCAO in C57BL/6 mice. qPCR data showed that both miR-132 and miR-212 were significantly increased in the penumbral area 24 h after MCAO compared with the sham group (Supplemental Fig. 1). Visual stimulation induces histone modification (acetyl-lys9 H3) on the CRE loci upstream of the miR-132 and miR-212 coding sequences and activates mature and primary miR-132 expression [8]. To examine the functional role of CRTC1 in the regulation of miR-132/212 in cerebral ischemia, we generated CRTC1 KO mice using CRISPR/Cas9, and Western Blot showed that CRTC1 expression was completely absent in the mouse (Fig. 1A). No differences were found between CRTC1 KO and WT mice in 8-week-old weight (Supplemental Fig. 2A), blood pressure (Supplemental Fig. 2B), and CBF under resting conditions (Supplemental Fig. 2C). qPCR showed no significant change in miR-132/212 expression in the nonischemic brain (Fig. 1B, C), while a significant reduction in miR-132/212 expression was detected in the CRTC1 KO penumbral area compared with WT 24 h after focal cerebral ischemia (Fig. 1D, E).

### CRTC1 deficiency aggravates ischemic brain injury

**Fig. 2:**
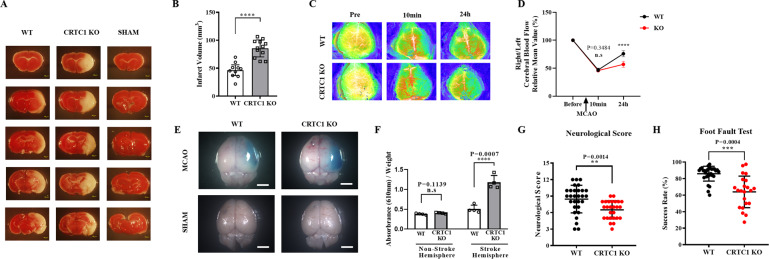
CRTC1 deficiency exaggerates blood–brain barrier breakdown and mice forelimb recovery after ischemia. **A** Representative TTC staining images of CRTC1 KO and WT mice brain sections 24 h after MCAO. **B** Quantification of infarct volume of CRTC1 KO and WT mice. *n* = 10 for each group. **C** Cerebral blood flow (CBF) pictures of CRTC1 KO and WT mice before and after MCAO. **D** Quantification of relative CBF change of right/left MCA responsible area. *n* = 10 for each group. Representative images (**E**) and quantification (**F**) of Evans Blue extravasation in CRTC1 KO and WT mice brains after MCAO. *n* = 4 for each group. Mice behavior deficits 24 h after MCAO were examined by Neurological Score (**G**) and Foot fault Test (**H**). *n* = 29 for WT, *n* = 26 for CRTC1 KO.

To clarify the role of CRTC1 in ischemic stroke, we subjected both CRTC1 KO and WT mice to 60 min of MCAO and evaluated the infarct volume using TTC staining after 24 h (Fig. 2A). Strikingly, the CRTC1 KO mice showed an approximately twofold increase in ischemic stroke volume (Fig. 2B) compared with WT mice. Consistent with this finding, CRTC1 KO mice exhibited a twofold higher death rate compared with WT mice 24 h after MCAO (Supplemental Table 1). We also monitored cerebral blood flow (CBF) reperfusion in both CRTC1 KO and WT mice (Fig. 2C and Supplemental Fig. 2C) and found although no significant CBF changes were found until 10 min after the operation, CBF reperfusion was worsened in CRTC1 KO mice compared with WT mice 24 h after MCAO (Fig. 2D). To determine whether this difference in CBF reperfusion was caused by BBB breakdown, we examined BBB integrity using Evans Blue dye (Fig. 2E and Supplemental Fig. 3). Compared with the nonischemic hemisphere, there was an increase in Evans Blue staining in the ischemic hemisphere in both CRTC1 KO and WT mice, but the accumulation of Evans Blue was greater in the CRTC1 KO compared with the WT (Fig. 2F). These results suggest that CRTC1 deletion increases ischemic stroke volume and worsens BBB leakage induced by MCAO.

Next, we examined the effect of CRTC1 knockout on mouse behavioral outcomes after stroke. Both CRTC1 KO and WT mice exhibited significant functional deficits after MCAO (Fig. 2G, H). Compared with WT mice, CRTC1 KO mice showed a statistically worse neurological score (Fig. 2G), and significant worse forelimb use in both the foot fault test (Fig. 2H). We also performed rotarod test and cylinder test for behavior assessment, but failed to distinguish differences. This may because that mice showed extremely weakness for adherence to drum in the rotarod test, and barely spontaneous forelimb use for vertical movement in cylinder test. This is consistent with a previous report [24]. These results suggest that CRTC1 deletion aggravates ischemic stroke outcome, without altering cerebral anatomical structures.

### CRTC1 deficiency and miR-132 knockdown aggravate neuronal damage via RBFox-1

**Fig. 3:**
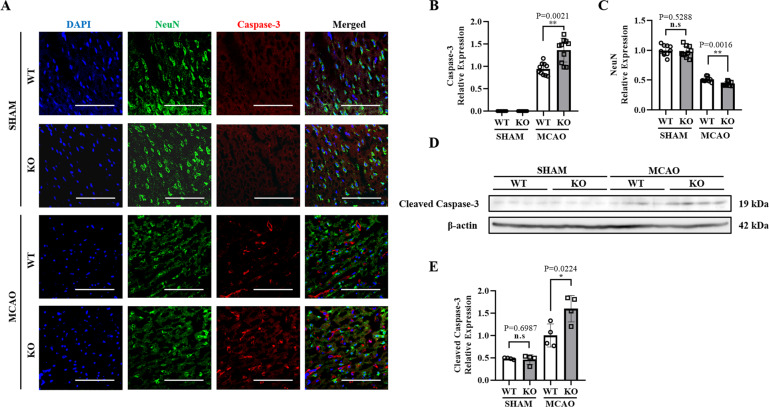
CRTC1 deletion and miR-132 knockdown aggravate neuron apoptosis. Representative immunostaining images (**A**) and quantification of Caspase-3 (**B)** and NeuN (**C)** in CRTC1 and KO brain sections. (Scale Bar, 100 μm). Representative western Blot image (**D**) and quantification (**E**) of cleaved caspase-3 in CRTC1 KO and WT penumbra regions.

The impact of CRTC1 deletion on stroke outcomes led us to hypothesize that CRTC1 affects NVU integrity by regulating miR-132/212. RBFox-1, which is reported to be critical for neuronal activity, was predicted to be a miR-132/212 target by bioinformatics analysis. In order to investigate neuron apoptosis in vivo, we applied NeuN and Caspase-3 immunostaining to CRTC1 KO and WT mice brain sections (Fig. 3A). We found CRTC1 KO mice showed an enhanced Caspase-3 expression (Fig. 3B) and a relatively decreased NeuN expression (Fig. 3C) compared to WT mice in cortex of penumbra area. Western Blot (Fig. 3D) showed an elevated cleaved Caspase-3 (Fig. 3E) in CRTC1 KO mice after MCAO, indicating a higher apoptosis in CRTC1 KO mice.

**Fig. 4:**
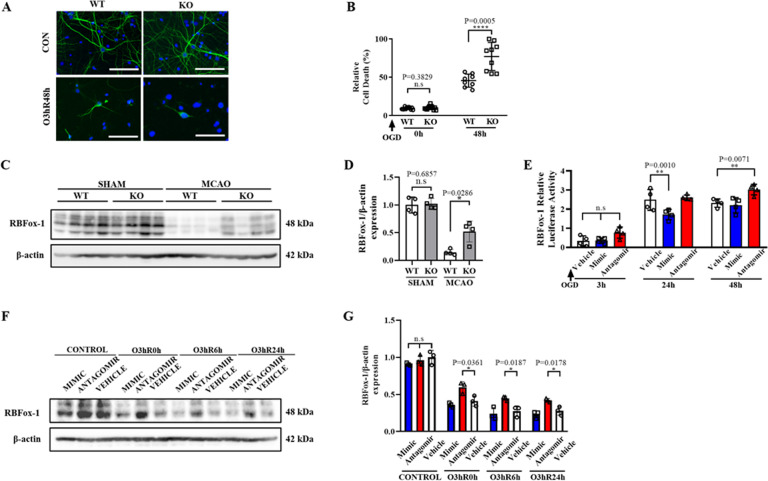
CRTC1 deletion and miR-132 knockdown aggravate neuron damage. **A** Representative microtubule-associated protein 2 (MAP2) immunostaining images of CRTC1 KO and WT neurons, before and after OGD. (Scale Bar, 100 μm). **B** LDH assay of CRTC1 KO and WT neuron up to 48 h after ischemia/reperfusion. Representative Western Blot images (**C**) and quantification (**D**) of RBFox-1 in CRTC1 KO and WT mice penumbra region. **E** Relative luciferase activity of CHO cell line co-transfected with RBFox-1 and miR-132 vehicle/mimic/antagomir. Representative Western Blot images (**F**) and quantification (**G**) of RBFox-1 in neurons transfected with miR-132 mimic/antagomir/vehicle, before and after OGD.

To further investigate the mechanism underlying damage induced by CRTC1 deletion in neurons, we subjected primary cortical neurons derived from CRTC1 KO and WT mice to Oxygen-glucose deprivation (OGD). The CRTC1-deficient neurons displayed significantly exacerbated ischemic damage compared with wild type, as shown by MAP2 immunostaining (Fig. 4A) and LDH assay (Fig. 4B). We examined the expression of RBFox-1 in both CRTC1 KO and WT mice under SHAM and ischemic conditions (Fig. 4C). Although no significant difference of RBFox-1 expression was observed between CRTC1 KO and WT mice under SHAM condition, MCAO induced RBFox-1 expression decrease in both CRTC1 KO and WT mice, while CRTC1 deficiency attenuated this decrease (Fig. 4D). To investigate whether RBFox-1 mRNA is regulated by miR-132 after ischemia, we generated a CHO line stably expressing the RBFox-1 3′-UTR (Fig. 4E) and transfected this cell line with miR-132 mimic, antagomir, and vehicle. We observed that miR-132 mimic significantly downregulated RBFox-1, while miR-132 antagomir transfection attenuated this decrease, especially after OGD/Reperfusion in CHO cells stably expressing the RBFox-1 3′-UTR. This suggests that miRNA-132 regulates RBFox-1 mRNA under ischemic conditions. To date, there are no published reports of RBFox-1-mediated splicing changes after cerebral infarction. Here, we show, for the first time, changes in RBFox-1 after stroke, both in vivo and in vitro, and the role of CRTC1-miR-132/212 in these changes. qPCR revealed no significant changes in RBFox-2 or RBFox-BS in mouse brains (Supplemental Fig. 4). Furthermore, to confirm that the RBFox-1 expression change was caused by miR-132/212, we transfected neurons with miR-132 mimic, antagomir or vehicle, and then subjected these cultures to OGD, with or without subsequent reperfusion (Fig. 4F). No statistically significant differences in RBFox-1 expression were observed among these groups under control conditions. Antagomir significantly repressed the inhibition by miR-132 on RBFox-1 expression, while mimic slightly promoted the inhibition compared to the vehicle group after OGD/Reperfusion (Fig. 4G). Taken together, these results suggest that CRTC1 deletion and miR-132/212 knockdown both aggravate post-stroke neuronal damage and upregulate RBFox-1 in neurons.

### CRTC1 deficiency suppresses vascular bed and destabilizes tight junction protein expression in the ischemic mouse brain

**Fig. 5:**
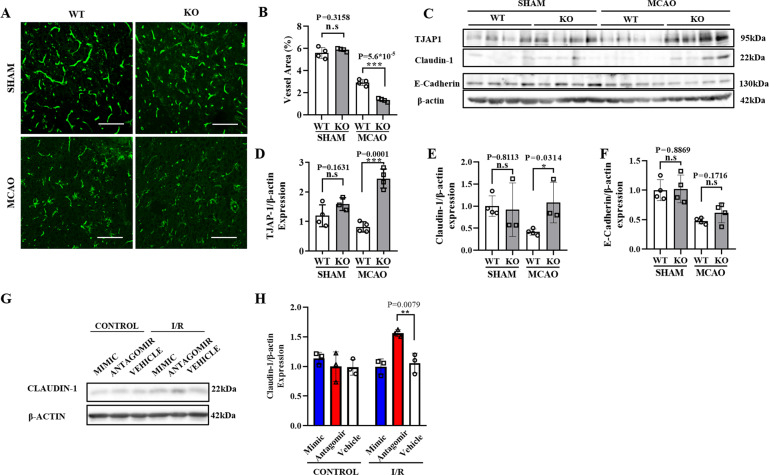
CRTC1 deficiency suppresses vascular bed and destabilizes tight junction proteins in ischemic mice brains. Representative images (**A**) and quantification (**B)** of *Lycopersicon esculentum* Lectin immunofluorescence staining of mice penumbra (Scale Bar, 100 μm). Representative western blot images (**C**) and quantification of TJAP-1 (**D**), Claudin-1 (**E**), and E-Cadherin (**F**) expression after MCAO. Representative Western Blot images (**G**) and quantification (**H**) of Claudin-1 in mouse brain microvascular endothelial cells (BMECs) transfected with miR-132 mimic/antagomir/vehicle, before and after OGD.

To investigate whether CRTC1 deletion leads to endothelial cell dysfunction, we performed *Lycopersicon esculentum* lectin immunofluorescence staining to examine CRTC1 KO and WT brain vascular formation (Fig. 5A). No statistically significant change was observed in penumbra between the two groups under control conditions, and cerebral ischemia led to vasculature damage in the penumbra in both CRTC1 KO and WT mice. However, CRTC1 KO mice exhibited aggravated vascular bed damage compared with WT (Fig. 5B).

In the adult mammalian brain, interactions among several tight junction proteins, such as claudin-5 and adhesive junction protein E-Cadherin, contribute to BBB integrity. To examine the mechanism underlying endothelial damage induced by CRTC1 deletion, we performed western blot for tight junction proteins in CRTC1 KO and WT penumbra. TJAP-1, which is demonstrated to be critical for BBB integrity, was also evaluated (Fig. 5C). In the acute phase after stroke, CRTC1 KO mice exhibited a statistically significant increase in TJAP-1 expression (Fig. 5D) and Claudin-1 expression (Fig. 5E) in comparison with WT mice. We also examined E-cadherin expression (Fig. 5F) in CRTC1 KO and WT brain, but no significant differences were observed. We next examined tight junction protein expression in mouse brain microvascular endothelial cells (BMECs) transfected with miR-132 mimic, antagomir, or vehicle, before and after OGD (Fig. 5G). An overall upregulation of Claudin-1 was observed after OGD/reperfusion, while antagomir increased specially Claudin-1 expression and mimic reduced slightly Claudin-1 expression compared to the vehicle group (Fig. 5H). These results suggest that miR-132 regulates Claudin-1 mRNA under ischemic condition.

**Fig. 6:**
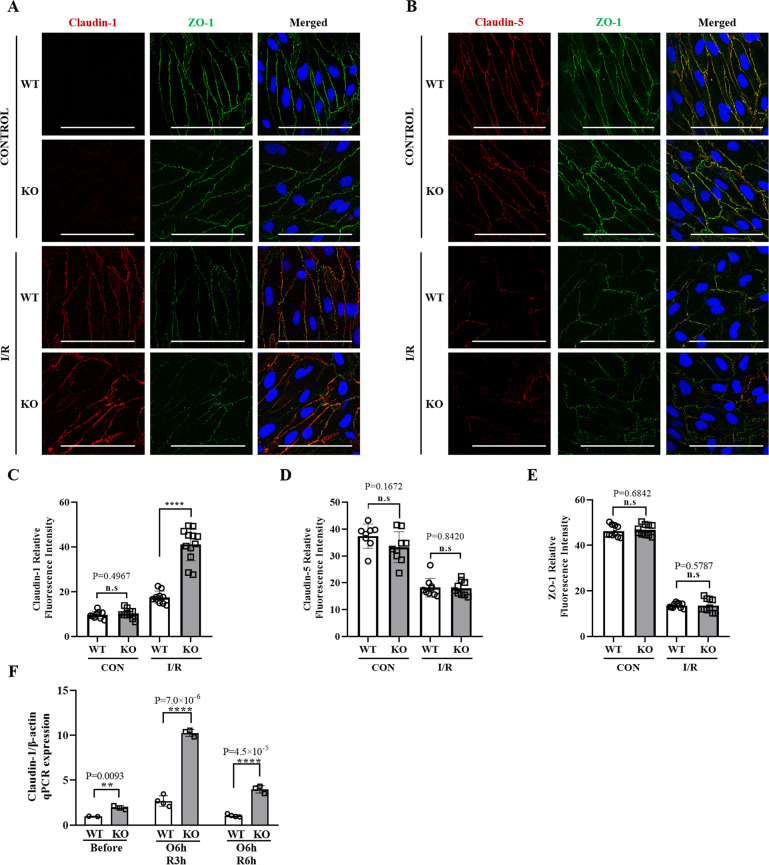
CRTC1 deficiency enhances TJ proteins destabilization induced by ischemia/reperfusion in Mouse BMECs. Representative immunostaining images of Claudin-1/ZO-1 (**A**), Claudin-5/ZO-1 (**B**) and quantification of Claudin-1 (**C**), Claudin-5 (**D**), and ZO-1 (**E**) of BMECs derived from CRTC1 KO and WT, before and after ischemia/reperfusion (Scale Bar, 100 μm). **F** qPCR results of Claudin-1 in BMECs derived from CRTC1 KO and WT mice, before and after ischemia/reperfusion. *n* = 4 for each group.

In CRTC1 KO-derived BMECs, the expression of Caludin-1 mRNA was significantly higher than that in the wild type, especially after OGD/Reperfusion. Next, we performed immunofluorescence staining for Claudin-1, Claudin-5, and ZO-1 in BMECs, under control and ischemic conditions (Fig. 6A, B). Ischemia dramatically increased Claudin-1 expression in BMECs, while KO BMECs showed an enhanced upregulation of Claudin-1 compared with WT (Fig. 6C). We also evaluated the Claudin-5 (Fig. 6D) and ZO-1 (Fig. 6E) expression in BMECs, but no statistically significant differences were found between KO and WT, before or after OGD. To further elucidate the regulatory role of miR-132/212 in endothelial cells, we transfected Human umbilical vein endothelial cells (HUVECs) with miR-132 mimic, antagomir, or vehicle, before or after OGD (Supplemental Fig. 5). Claudin-1 was not detected in HUVECs before or after OGD, and miR-132 transfection did not statistically alter Claudin-5 or ZO-1 expression compared with the control group. To elucidate the functional role of CRTC1 in tight junction protein expression, Claudin-1 qPCR was performed in BMECs derived from both wild-type and CRTC1 KO mice after ischemia (Fig. 6F). qPCR showed Claudin-1 expression was obviously elevated 3 h after OGD in both CRTC1 KO and wild-type BMECs. Collectively, these data suggest that CRTC1 deletion and miR-132/212 inhibition both increase Claudin-1 and TJAP-1 expression in endothelial cells after ischemia.

### MiR-132/212 translationally enhances BBB integrity in ischemia

**Fig. 7:**
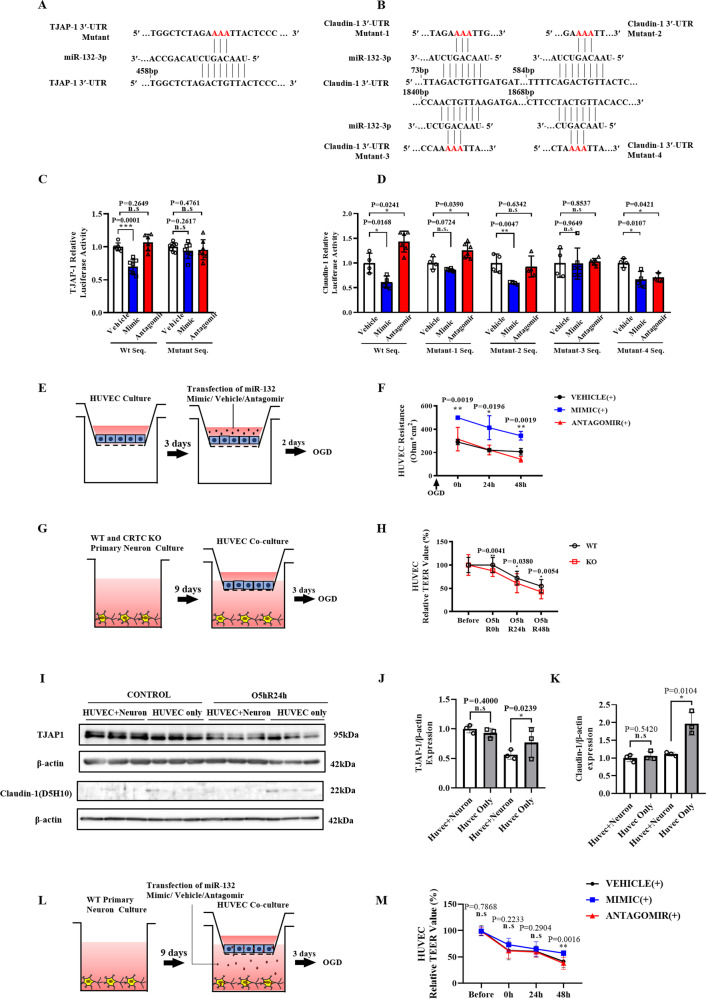
Neuron secretes miR-132/212 to endothelial cells to alleviate BBB breakdown after ischemia/reperfusion. The partial sequence of miR-132 and its one putative binding site in the 3′-UTR of the mouse TJAP-1 (**A**) and four putative binding sites in the 3′-UTR of the mouse Claudin-1 (**B**), with each mutant 3′-UTR. Quantitative data showed that transfection of miR-132 mimic decreased luciferase activity of the reporter vector containing wild-type 3′-UTR sequence of mouse TJAP-1 (**C**) and Claudin-1 (**D**), while transfection of miR-132 mimic or antagomir had no effects on the luciferase activity of the reporter vector containing mutation of 468-470 bp within mouse TJAP-1 3′-UTR (**C**) and mutation of 1844-1846 bp within mouse Claudin-1 3′-UTR (**D**). (**E**) HUVECs were transfected with miR-132 mimic/vehicle/antagomir, then subjected to OGD. (**F**) TEER evaluation indicates that miR-132 mimic significantly attenuated the decline in TEER values after OGD, while miR-132 antagomir aggravated HUVEC resistance to hypoxia. *n* = 3 for each group. (**G**) A BBB model in vitro was constructed by a co-culture system of HUVECs and neurons derived from CRTC1 KO and WT mice. (**H**) HUVECs co-cultured with CRTC1 KO neurons exhibited progressively decreased TEER values after OGD compared with HUVECs co-cultured with WT neurons. Representative Western Blot images (**I**) and quantification of TJAP-1 (**J**) and Claudin-1 (**K**) of HUVECs co-cultured with or without neurons, before and after OGD. (**L**) HUVECs were co-cultured with neurons that were transfected with miR-132 mimic/antagomir/vehicle, then subjected to OGD. (**M**) TEER evaluation indicates that miR-132 mimic by paracrine secretion significantly attenuated the decline in ECs TEER values after OGD. *n* = 10 for each group.

To clarify the mechanism by which miR-132/212 regulates TJAP-1 and Claudin-1 expression, we conducted bioinformatics analysis and dual-luciferase assay. Bioinformatics analysis indicated one conserved miR-132/212 binding site within the 3′-UTR of mouse TJAP-1 mRNA (Fig. 7A) and four conserved miR-132/212-binding site within the 3′-UTR of mouse Claudin-1 mRNA (Fig. 7B), suggesting that miR-132/212 might translationally repress these tight junction proteins by direct binding to their 3′-UTR mRNAs. Therefore, we transfected pmiRGLO vector containing each 3′-UTR sequence into CHO cells to generate a stable cell line expressing 3′-UTR of these genes (wild-type or mutant type), then co-transfected these CHO cells with miR-132 mimic, miR-132 antagomir or vehicle. We found transfection of miR-132 mimic into CHO significantly decreased the luciferase activity of the wild-type 3′-UTR of mouse TJAP-1 and Claudin-1, while transfection of miR-132 antagomir into CHO significantly increased the luciferase activity of the wild-type 3′-UTR of mouse Claudin-1 (Fig. 7C, D). Importantly, neither miR-132 mimic nor antagomir transfection showed any statistically significant effects on the luciferase activity in CHO that contains mutation of 468–470 bp within mouse TJAP-1 3′-UTR and that contains mutation of 1844–1846 bp within mouse Claudin-1 3′-UTR. Collectively, these findings suggest miR-132 specifically suppress TJAP-1 and Claudin-1 expression by directly binding to their 3′-UTR mRNA regions. To investigate the role of miR-132/212 in endothelial cell function in ischemia, we measured the transendothelial electrical resistance (TEER) values of HUVECs transfected with miR-132 mimic, antagomir or vehicle, followed by OGD and up to 48 h of reperfusion (Fig. 7E). MiR-132 mimic significantly attenuated the decline in TEER values after OGD in comparison with vehicle (Fig. 7F).

**Fig. 8:**
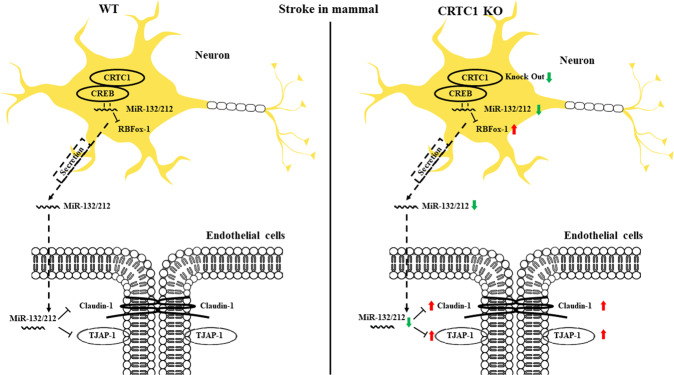
Graphic abstract. CRTC1-mediated miR-132/212 attenuates neuron damages via suppressing RBFox-1, and alleviates blood–brain barrier dysfunction by regulating TJAP-1 and Claudin-1 expression in ischemic stroke.

As described in previous studies, both CRTC1 and miR-132/212 are selectively expressed in neurons. Previous studies show that neurons secrete miR-132 to endothelial cells in zebrafish under ischemic stroke [25]. We observed that miR-132 levels in BMECs were not different between wild-type and CRTC1 KO, before and after OGD (Supplemental Fig. 6A). Western blotting suggested that CRTC1 is abundantly expressed in neurons compared with endothelial cells (Supplemental Fig. 6B). To elucidate the functional role of miR-132/212 in the BBB in ischemia, we modeled the BBB in vitro, using a co-culture system of vascular endothelial cells (HUVECs) and neurons derived from CRTC1 KO or WT mice (Fig. 7G). We assessed the endothelial cells’ resistance to OGD by TEER, up to 48 h after reperfusion. HUVECs co-cultured with CRTC1-deficient neurons exhibited a progressively exacerbated barrier breakdown after OGD, compared with HUVECs co-cultured with WT neurons (Fig. 7H). To specify the direct relationship between neuron-derived miR-132 and TJPs, we seeded HUVECs in six-well transwell inserts, co-cultured with or without primary neurons. Cultures were subjected to 3 h OGD following 24 h reperfusion, then applied to Western Blot assay (Fig. 7I). After 24 h reperfusion following 3 h OGD, HUVEC co-cultured with neurons showed a relatively lower expression of TJAP-1 (Fig. 7J) and Claudin-1 (Fig. 7K) than HUVECs cultured alone. To functionally test the paracrine secretion of miR-132 from neuron, we transfected miR-132/212 mimic/antagomir/vehicle into neurons which were co-cultured with HUVECs, then subjected the cultures to OGD following up to 48 h reperfusion (Fig. 7L). No significant differences were found before OGD among these groups. After subjected to OGD/Reperfusion, HUVECs co-cultured with neurons that were transfected with miR-132 mimic gradually attenuated the TEER decline compared to the vehicle group, suggesting that miR-132 upregulated ECs resistance to OGD by an external pathway. HUVECs co-cultured with neurons that were transfected with antagomir showed hardly differences compared to the vehicle group, which may because OGD/Reperfusion inducing ECs damage was already occurred that could be hardly exaggerated furthermore (Fig. 7M). Taken together, these results indicate that CRTC1 affects BBB integrity by a paracrine pathway, suggesting that CRTC1-mediated release of miR-132/212 from neurons impacts the functional integrity of vascular endothelial cells (Fig. 8).

## Discussion

Maintaining BBB integrity is vitally important for therapies of cerebral ischemia. Here, we investigated the functional role of miR-132/212 in neuronal/endothelial cell injury in cerebral ischemia. We found that CRTC1 affects miR-132/212 expression in mice, and we showed that miR-132 within the NVU regulates BBB homeostasis through an intercellular mechanism. In this study, we demonstrated that CRTC1 KO mice display a progressive downregulation of miR-132/212 in the early post-stroke phase, and exhibit aggravated neurological deficits and ischemic cerebral damage. Considering that our previous study identified CRTC1 regulated neuron survival via BDNF and PGC-1α [12], here we found that CRTC1-modulated miR-132/212 protects against neuronal injury by modulating RBFox-1. Furthermore, miR-132/212 released from neurons enters endothelial cells and acts on tight junction proteins, such as Claudin-1 and TJAP-1.

MiR-132/212 is a well-studied miRNA cluster in CNS disorders such as neurogenerative diseases [26] and intracerebral hemorrhage [27]. CREB-regulated transcription coactivator (CRTC) includes CRTC1, CRTC2, and CRTC3, among which CRTC2 has been demonstrated to dictate the vascular endothelial function [28], and CRTC1 is a specifically expressed protein in neuron. CRTC1 has been suggested to act as a critical role in several neurodegenerative diseases, such as Alzheimer’s disease [29–31], and Huntington’s disease [32, 33]. Nucleus-specific CRTC1 activation in the hippocampus enhances contextual fear memory [34]. In this study, we predicted that miR-132/212 was regulated by CRTC1. Consistent with this hypothesis, we found that CRTC1 deficiency reduced levels of miR-132/212 under ischemic conditions.

As several targets and signaling pathways may participate in the function of miR-132/212 in ischemia, we focused on the role of miR-132/212 on neuron–endothelial interaction and neurological deficits after cerebral ischemia. Alternative pre-mRNA splicing has a critical role in CNS gene expression. The RBFox proteins regulate the splicing of numerous transcripts in neurons, thereby affecting synaptic transmission and membrane stability [35]. Our findings show that miR-132/212 inhibits RBFox-1 expression, but does not alter RBFox-2 or RBFox-3. To clarify whether the upregulation of RBFox-1 results from miR-132/212 inhibition, we transfected primary cortical neurons with miR-132 mimic or antagomir and subjected them to OGD. Inhibition of miR-132 was sufficient to significantly increase RBFox-1 expression.

BBB is a highly complex and dynamic structure that stringently regulates substance exchange by intricate interactions of tight junction proteins between endothelial cells [36]. Recently, Zuo et al. reported that miR-132 promotes BBB integrity by repressing MMP9 via directly binding to its 3′-UTR and increasing MMP9 downstream tight junction proteins which refer to VE-Cadherin and β-catenin, but not differing TJPs like Occludin. It should be noted that TJPs in our study refer to Claudin-1, Claudin-5, and TJAP-1, that are different from the TJPs from the former study. The tight junction complex is mainly constructed by the linear polymers, which are formed by interactions of Claudin proteins with each other [37, 38]. ZO-1 is a scaffold protein essential for Claudins polymerization. Recently, increasing studies suggested that rather than the expression level change of one key TJ protein, but a destabilized organization of TJ complex is more responsible for BBB breakdown [39], such as Claudin-1 knockdown led to a dynamic TJ proteins destabilization and improve BBB permeability and functional recovery after stroke [23]. In addition to TJ, adherens junctions (AJs) complex is also a major junctional complex in BBB. AJs are composed primarily of cadherin proteins that mainly contribute to cell–cell adhesion and stability by linking to the actin cytoskeleton via β-catenin proteins [40, 41]. β-Catenin signaling in endothelial AJ plays a crucial role in maintaining cellular polarity and is important for maintaining adult BBB integrity in stroke [42]. However, the precise role of AJ in BBB and the relationship between AJ and TJ in neurological disorders have not yet been elucidated, so further studies are needed. TJAP-1 is a peripheral membrane protein located in TJ complexes first reported in 2001 [43], but its function remains unclear. This study is consistent with the previous studies. Moreover, our work demonstrated that CRTC1 deficiency contributes to BBB destabilization by increasing Claudin-1 expression in BMEC after ischemia/reperfusion for the first time using immunofluorescence staining, and that inhibition of Claudin-1 by miR-132 improves BBB recovery after ischemia/reperfusion. We clarified the regulation of miR-132 on Claudin-1 by insert mutation into 1844–1846 bp within Claudin-1 3′-UTR. Although its role in BBB remains unclear, TJAP-1 is presumed to have a regulatory role in BBB integrity because of its localization toward the cytoplasmic face of TJs and within the Golgi stack [44]. In this study, TJAP-1 and Claudin-1 exhibited a significant increase in CRTC1 KO mice compared with WT mice, leading to BBB damage by Claudin-5/Claudin-1 dysregulation in the CRTC1 KO brain after stroke. Furthermore, our observation of HUVECs transfected with miR-132 mimic/antagomir revealed that miR-132 directly regulates Claudin-1 and TJAP-1 in endothelial cells. Using TEER, we found that transfection of miR-132 antagomir significantly reduced the resistance of HUVECs to hypoxia, indicating that miR-132 directly regulates endothelial cell interactions.

Next, as both CRTC1 and miR-132/212 are neuron-specifically expressed, we investigated whether intercellular interaction between neurons and endothelial cells maintain neurovascular homeostasis. BBB functions depend mostly on the perivascular microenvironment, especially on neuronal cells and BBB-forming endothelial cells. Under ischemic conditions, various factors are released from neurons to endothelial cells, in an attempt to attenuate BBB dysfunction. We investigated the role of miR-132/212 in neuron–endothelial cell interactions using a co-culture system. Consistent with the evidence that neurons secrete exosomes containing miR-132/212 to endothelial cells in zebrafish [25], we found that HUVECs cultured with CRTC1 KO neurons, which lack miR-132/212, showed reduced resistance to hypoxia. This suggests that CRTC1 in neurons regulates TJ protein interactions in endothelial cells via a paracrine signaling mechanism to maintain BBB integrity in Neurovascular unit after stroke.

The CRTC family was initially identified as co-activators of CREB via the dZIP domain, independent of the CBP/p300 pathway [45, 46]. Unlike CRTC2 and CRTC3, CRTC1 is extremely highly expressed in the brain, and its expression level far exceeds that of CREB [47–49]. This suggests that CRTC1 has important and diverse functions in the brain [50].

Taken together, our findings demonstrate that CRTC1 deficiency aggravates neurological deficits in mice in the early phase after stroke. This worsening is mediated by RBFox-1 via disruption of CRTC1-miR-132 signaling in neurons. The miR-132/212 dysregulation impairs BBB function and is associated with perturbations in TJAP-1, Claudin-1, and Claudin-5. Further studies are needed to clarify the paracrine communication mechanisms by which miRNAs and non-coding RNAs affect CNS pathology after stroke. Investigation of the intercellular signaling mechanisms mediated by miRNAs may lead to novel approaches for the treatment of stroke.

## Materials and methods

### Animal

CRTC1 knockout was conducted with CRISPR/Cas9 on C57BL/6 background at Osaka University Genome Editing Research and Development (R&D) Center [51]. Details were described in the Supplementary materials. Animals were raised under standard conditions of light (lights on: 8:00 a.m. to 8:00 p.m.) and temperature (23 °C, 40% humidity). Systolic blood pressure was measured on 8 weeks old by a blood-pressure monitor (SOFTRON, Tokyo, Japan).

### Transient focal cerebral ischemic model

Transient focal cerebral ischemia was conducted in 8–12 weeks old male mice by MCAO (MCAO) as described in our previous study [52]. Mice were randomly divided into SHAM and MCAO groups. General anesthesia was performed using isoflurane with an open mask, and cortical CBF was monitored by a laser-Doppler flowmetry from before to 10 min after operation. The right middle cerebral artery was occluded for 60 min with a suture followed by 24 h of reperfusion. Only mice with less than 30% of baseline control microperfusion during the first minute of occlusion were used in subsequent experiments. After the operation, mice recovered in their individual cages normally.

### Measurement of cerebral blood flow (CBF)

The measurement of CBF was conducted as previously described [53]. Surface CBF was recorded by a laser speckle blood flow imaging system (Omegazone OZ-1). After general anesthesia, the skull was exposed by a midline scalp incision. The surface of the skull was wiped clean with saline-soaked gauze before recording. Color-coded CBF images were obtained in high-resolution mode. The mean CBF value was measured in identically sized regions of interest (900 pixels) located 3 mm posterior and 2.5 mm lateral from the bregma [53].

### 2,3,5-triphenyltetrazolium hydrochloride (TTC) staining

After 24 h of reperfusion, the brains were collected and sectioned at 2-mm intervals, and then stained with 2% TTC at room temperature for 1 h. The brain sections were fixed in 4% PFA for 30 min, and photographed with a digital microscope (Olympus SZX12). The infarct volume was calculated as: whole contralateral hemisphere volume − nonischemic ipsilateral hemisphere volume.

### Evans Blue extravasation

After 24 h of MCAO, 100 μl of 4% Evans Blue was injected as a tracer into the circulation via the caudal vein. After 10 h of reperfusion, the brain was collected, and photographed before and after sectioned with a digital microscope (Olympus SZX12). The hemispheres were collected and homogenized respectively in 500 μl formamide (Nacalai Tesque, Kyoto, Japan) at 60 °C overnight. Supernatants were collected, and 100 μl aliquots were used for absorbance detection at 610 nm. Formamide incubated at 60 °C overnight was used as the blank, and the OD of each mouse was calculated as: average absorbance/hemisphere weight.

### Quantitative real-time PCR analysis

For the miR-132/212 assay, total RNA, including small RNA, was extracted from neurons or tissues using the mirVana miRNA Isolation Kit (Thermo Fisher Scientific, Waltham, MA, USA). cDNA was prepared by reverse transcription (RT) from 1 μg of total RNA using Taqman MicroRNA Reverse Transcription kit (Thermo Fisher Scientific). The RT products were used in real-time PCR analysis with the Taqman MicroRNA assay for miR-132 (Mm04238115_s1) and miR-212 (Mm04238228_s1), using the Taqman Fast Advanced Master Mix (Thermo Fisher Scientific). U6 RNA was used as normalization.

For relative mRNA transcript quantification, cDNA was prepared from 1 μg total RNA using the SuperScript VILO cDNA Synthesis Kit (Invitrogen, Waltham, MA, USA). Power SYBR Green PCR Master Mix (Thermo Fisher Scientific) was used for real-time PCR. β-actin and 36B4 served as endogenous control. Primer sequences were described in Supplemental Table 2. Relative expression was calculated using the comparative CT method with the 7900HT Real-Time PCR System (Applied Biosystems, Waltham, MA, USA).

### Behavioral test

The criteria used for neurological score were described in a previous study [54]. Spontaneous activity, symmetry of movements, symmetry of forelimbs, climbing wall of wire cage, reaction to touch on either side of trunk, and response to vibrissae touch were scored as 0–3 points, respectively. The lower score indicates worse neurological deficits. For the foot fault test, mice were placed on an elevated gridded platform above the surface and allowed to walk for 5 min. A foot fault was noted when the left forefoot misstepped and fell through the space between the grids. The percentage of left foot faults was measured for statistical analysis during the whole observation. For the cylinder test, mice were placed inside a transparent cylinder with a tri-fold mirror placed behind to ensure both forelimbs could be seen. Forelimb use for vertical exploration was evaluated by counting left forelimb contacts on the cylinder. The success rate was obtained as: right forelimb failed contact/right forelimb contact + left forelimb contact + both contacts. For the rotarod test, the time at which mice fell off the drum was recorded as latency to fall. Mice alive but showed no spontaneous activity were excluded for the behavior test.

### Cell cultures and transfection

Primary cultures of cortical neurons were prepared as described previously [55]. Briefly, neuronal cultures were prepared from the cortex of embryonic day 16 (E16) mouse embryos. Cortical tissue was incubated in dissection medium (Dulbecco’s modified Eagle’s medium containing 100 U of papain and 0.5 mg/ml DNase type II) for 30 min at 37 °C. After centrifugation, cells were plated onto 24-transwell plates, 12-transwell plates, 6-transwell plates, 4-well culture slides, 60-mm dishes, or 4-chamber glass slides (Corning, NY, USA) coated with polyethylenimine. Cells were cultured to a final concentration of 7.0 × 10^5^ cells/ml in high-glucose DMEM (Wako, Osaka, Japan) containing 10% fetal calf serum (Invitrogen) and 100 IU/ml penicillin. After 24 h, the medium was changed to Neurobasal medium (Invitrogen) supplemented with B-27 (Invitrogen). The cells were cultured at 37 °C in a humidified atmosphere of 95% air and 5% CO_2_ and used after 10–11 days in vitro when most cells displayed a neuronal phenotype. HUVECs were purchased from PromoCell (Heidelberg, Germany), and were cultured following the manufacturer’s instructions.

BMEC isolation and purification were performed as described previously. Briefly, brain tissues were isolated from 3-week-old CRTC1 KO and WT mice and dissociated with 1 mg/ml collagenase type2 (Worthington, Columbus, OH, USA). Then 20% BSA (Wako) in DMEM/F-12 (Wako) was used to remove myelin. The cell suspensions were incubated with 1 mg/ml collagenase/dispase (Roche, Basel, Switzerland), and then gently injected onto Percoll (GE Healthcare, Chicago, IL, USA) for centrifugation at 1000 × *g* for 10 min. The endothelial cell layer was collected and cultured in DMEM/F-12 medium.

MiR-132 mimic, vehicle, and antagomir were purchased from Bioneer Cooperation (Daejeon, Korea). All procedures were conducted strictly as the manufacturer’s instructions.

### Oxygen-glucose deprivation (OGD)

OGD was conducted by placing cultures in an anaerobic atmosphere as previously described. Briefly, cultures were washed with phosphate-buffered saline (PBS) and incubated in glucose-free Earle’s balanced salt solution (EBSS) (Biological Industries, Israel) in an anaerobic environment of 95% N_2_/5% CO_2_, maintaining an O_2_ pressure of 10–15 Torr, at 37 °C for 2 or 3 h. After incubation, OGD was terminated by replacing with complete medium and returning the cultures to a normoxic chamber.

### Cell viability assay

To evaluate neuronal death, the Cytotoxicity Detection Kit (Roche) was used with the lactate dehydrogenase (LDH) assay at 0, 24, or 48 h after termination of OGD. For each culture, 200 μl of supernatant was transferred to a 96-well plate. After a brief centrifugation at 4 °C, 400 × *g* for 10 min, 50 μl of supernatant was transferred from each well to a new 96-well plate for the next step. The cytotoxicity detection buffer was mixed according to the manufacturer’s instructions, and 50 μl of mixture was added to the corresponding well using a multichannel pipette. The plate was wrapped with aluminum foil and incubated at room temperature for 30 min. The OD was measured with an absorbance microplate reader (CORONA SH-9000Lab) at 490 and 650 nm. Neuronal cytotoxicity was calculated as: LDH release (OD_490_)/LDH release (OD_650_). One hundred percent cell death rate was revised to 2% TritonX-100 (Wako) pre-added in neuronal cultures before supernatant collection. Background control was recorded as absorbance value of 100 μl cytotoxicity detection buffer mixture. Neuron death rate % was calculated as (Example value – Background control)/(Maximum LDH release—Background control) [12].

### Immunofluorescence staining

For immunofluorescence staining in vitro, neurons and HUVECs were cultured in four-well slides. Cultures were washed and fixed with 4% PFA at room temperature for 30 min. Slides were washed three times with PBS and then incubated with 0.3% TritonX-100 at room temperature for 30 min. After 30 min of blocking with 10% donkey serum in TBS, slides were incubated with the following primary antibodies: MAP2 (1:500; Invitrogen), Claudin-5 (1:500; Abcam), Claudin-1 (1:500; Invitrogen) and ZO-1 (1:500; Invitrogen). Alexa Fluor 488-labeled donkey anti-mouse IgG, 647-labeled donkey anti-rabbit IgG, and 488-labeled donkey anti-rat IgG (Invitrogen) were used as secondary antibody.

For immunofluorescence staining in vivo, freshly isolated brains were covered in sufficient OCT embedding compound on dry ice, and cut into 16-μm-thick sections. Sections were attached to slides and fixed with 1% PFA at room temperature for 10 min, and incubated with 0.1% TritonX-100 at room temperature for 10 min. After 30 min of blocking with 10% donkey serum in TBS, slides were incubated with 488 conjugated *Lycopersicon esculentum* tomato lectin (1:1000; Vector Laboratories, Burlingame, CA, USA) overnight at 4 °C in the dark. For immunofluorescence staining of NeuN and Caspase-3, mice were sacrificed after 2% PFA perfusion, and brains were collected and cut into 16-μm-thick sections. Sections were incubated with 0.1% TritonX-100 at room temperature for 30 min. After 30 min of blocking with 10% donkey serum in TBS, slides were incubated with NeuN (1:250; Novus Biologicals, Centennial, CO, USA) and Caspase-3 (1:250; Cell Signaling) overnight at 4 °C, then subjected to 3 h incubation with secondary antibody (1:500; Invitrogen) including Alexa 488-labeled donkey anti-Mouse IgG and Alexa 594-labeled donkey anti-Rabbit IgG. Both cells and brain sections were counterstained with DAPI (Vector Laboratories), and a confocal laser-scanning microscope (Zeiss LSM-710) was used for visualization to avoid interference between channels and saturation.

### Western blotting

Brains were collected in liquid nitrogen and lysed in TNE buffer on ice. Protein content was quantified with the BCA Protein Assay Kit (Thermo Fisher Scientific). Then, 10-μg protein samples were electrophoresed and transferred to PVDF membranes (Merck Millipore, Burlington, MA, USA). After 1 h of blocking with 5% non-fat dry milk, membranes were incubated with primary antibody overnight at 4 °C. After 1 h of secondary antibody incubation at room temperature, visualization was conducted with a luminoimage analyzer (ImageQuant LAS4000, GE Healthcare). For RBFox-1 normalization, membranes were subjected to WB Stripping solution (nacalai, Kyoto, Japan) after chemiluminescent exposure, then incubated with primary anti-β-actin antibody overnight at 4 °C. The gray value of each lane was measured with Image-J software, and normalized to β-actin to obtain protein expression. The following antibodies were used: CRTC1 (1:1000; Cell Signaling Technology, Danvers, MA, USA); TJAP-1 (1:1000; Novus Biologicals); RBFox-1 (1:1000; Merck Millipore); E-Cadherin (1:1000; Cell Signaling Technology); Claudin-1 (1:1000; Invitrogen); cleaved Caspase-3 (1:1000; Cell Signaling Technology); β-actin (1:5000; Proteintech, Rosemont, IL, USA).

### Dual-luciferase reporter assay

The miRNA databases miRBase (http://microrna.sanger.ac.uk) and TargetScan (http://www.targetscan.org) were used to identify potential miR-212/132 targets on BBB. the wild-type sequences of RBFox-1 3′-UTR, Claudin-1 3′-UTR, and TJAP-1 3′-UTR were constructed as Supplemental Table 3. Luciferase reporter genes were generated by inserting each 3′-UTR fragment downstream of the firefly luciferase gene in pmiRGLO, including TK-pRL (renilla) as an internal control. These pmiRGLO variants with mutations inserted into the miR-132/212 target sequences present in the 3′-UTR of TJAP-1 and Claudin-1 were obtained using a PrimeSTAR Mutagenesis Kit (Takara Bio, Shiga, Japan). Since the 3′-UTR of Claudin-1 has four assumed target sequences of miR-132, four mutants were made for each target sequence. The pmiRGLO vector containing each 3′-UTR fragment was transfected into CHO cells and a stable cell line expressing 3′-UTR of these genes was prepared by selection with G418. These stable CHO cell lines were co-transfected with miR-132 mimic, miR-132 antagomir, or vehicle (each 100 nM) using a NEPA21 electroporator (NEPA GENE). At 96 h post-transfection, the cells were subjected to a dual-luciferase assay (Promega, Madison, WI, USA) as previously described.

### Transendothelial electrical resistance (TEER)

A Millicell-ERS Volt-Ohm Meter was used, and all procedures were carried out as per the manufacturer’s instructions. To investigate the role of CRTC1 in the interaction between neurons and endothelium, co-culture of neurons and HUVECs was performed. Neuronal cultures were plated onto 24-well plates as described above. After 9 days, HUVECs were seeded onto inserts (SARSTEDT, Nümbrecht, Germany) placed on top of the well containing neurons, at a density of 10^4^ cells/well. After 3 days, the co-cultures were objected to OGD for 2.5 h. TEER was performed at 0, 24, and 48 h after OGD. To investigate the direct effect of miR-132 on endothelium, HUVECs were seeded onto inserts at a density of 10^4^ cells/well. Transfections of miR-132 mimic, vehicle or antagomir were performed as described above 3 days after seeding. HUVEC cultures were objected to OGD for 3 h, and TEER was performed at 0, 24, and 48 h after OGD.

### Statistical analysis

Comparisons between two separate groups were performed using unpaired non-parametric *t*-test. Multiple groups comparisons were performed using one-way ANOVA with Bonferroni’s post hoc test. All data are presented as means ± standard deviation (SD).

## Supplementary information

Supplemental Methods

Supplemental Tables

Supplemental Figure 1

Supplemental Figure 2

Supplemental Figure 3

Supplemental Figure 4

Supplemental Figure 5

Supplemental Figure 6

Supplemental Legends

## Data Availability

The datasets generated and analyzed during the current study are available from the corresponding author on reasonable request.
