# Supplementary material for: MiRNA-132/212 regulates tight junction stabilization in blood–brain barrier after stroke
Source: Cell Death Discov. 2021 Dec 8;7:380. doi: 10.1038/s41420-021-00773-w (PMC8654926; doi:10.1038/s41420-021-00773-w)
Supplement: Supplementary file 1 — Supplemental Methods [file 41420_2021_773_MOESM1_ESM.docx]

**Supplemental Materials**

**Expanded Methods**

**Generation of CRTC1 KO mice**

To generate CRTC1 KO mice, loxP sites were introduced on either side of exon 6 of CRTC1 by CRISPR/Cas9.

To design gRNAs, software tools (https://zlab.bio/guide-design-resources) predicting unique target sites throughout the mouse and rat genome were used. gRNAs were transcribed in vitro using a MEGAshortscript T7 Transcription Kit (Life Technologies) from synthetic double-stranded DNAs obtained from Integrated DNA Technologies. mRNA from pCas9-poly (ID #72602, https://www.addgene.org/CRISPR) was transcribed in vitro using a mMESSAGE mMACHINE T7 Ultra Kit (Life Technologies) from linearized plasmids and was purified using a MEGAClear kit (Life Technologies). lssDNAs were prepared by a simple method using nicking endonucleases as we previously reported^1^. Briefly, double-stranded DNA plasmids comprising a floxed allele, homology arms, and two nicking endonuclease sites were obtained from Thermo Fisher Scientific (GeneArt® Gene Synthesis). For digestion, 100 µg of the purified plasmid DNA was incubated at an optimum temperature for 2 to 3 h with nicking endonucleases, such as Nb.BbvCI and Nb.BbvCI (New England Biolabs). After purification by ethanol precipitation, the DNA was denatured with 3-fold amounts of formamide (Nacalai Tesque) at 80°C for 10 min, and then subjected to agarose gel electrophoresis. Bands corresponding to a single-strand DNA fragment were extracted using NucleoSpin® Gel and PCR Clean-up (Takara Bio). Finally, 2–4 µg of lssDNA was obtained using this method.

Pronuclear-stage C57BL/6 mouse embryos were prepared by thawing frozen embryos (CLEA Japan Inc.) For electroporation, 50–100 embryos at 1 h after thawing were placed into a chamber with 40 µL of Opti-MEM serum free media (Thermo Fisher Scientific) containing 400 ng/µL Cas9 mRNA, 100 ng/µL gRNA(up), 100 ng/µL gRNA(down) and 25 ng/µL lssDNA. They were electroporated with a 5 mm gap electrode (Nepa Gene) in a NEPA21 Super Electroporator (Nepa Gene). The electroporation pulses were voltage 225 V, pulse width 1 ms, pulse interval 50 ms, and number of pulses 4. The first and second transfer pulses were voltage 20 V, pulse width 50 ms, pulse interval 50 ms, and number of pulses 5. Mouse embryos that developed to the two-cell stage after the introduction of RNAs and lssDNA were transferred into the oviducts of female surrogates anesthetized with isoflurane (DS Pharma Animal Health).

The obtained CRTC1 heterozygotes were mated with C57BL/6J mice for 10 generations, and then bred and used for this study. Animals were raised under standard conditions of light (lights on: 8:00 a.m.–8:00 p.m.) and temperature (23 °C, 40% humidity).

**References**

1. Yoshimi K, Kunihiro Y, Kaneko T, et al. ssODN-mediated knock-in with CRISPR-Cas for large genomic regions in zygotes. Nat Commun 2016; 7: 10431. 2016/01/21. DOI: 10.1038/ncomms10431.
