# Supplementary material for: MiRNA-132/212 regulates tight junction stabilization in blood–brain barrier after stroke
Source: Cell Death Discov. 2021 Dec 8;7:380. doi: 10.1038/s41420-021-00773-w (PMC8654926; doi:10.1038/s41420-021-00773-w)
Supplement: Supplementary file 2 — Supplemental Tables [file 41420_2021_773_MOESM2_ESM.docx]

**Supplemental Table 1. Mortality rate of Mice**

|  | WT | CRTC1 KO |
| --- | --- | --- |
| Mice subjected to MCAO | 93 | 116 |
| Mice alive  (24 h) | 71 | 67 |
| Mortality rate | 23.66% | 42.24% |

**Supplemental Table 2. Primers sequences**

| RBFox-1 | Fwd | 5ʹ-AGACCACTGTCCCTGACCAC-3ʹ |
| --- | --- | --- |
|  | Rev | 5ʹ-CATTTGTCGGAGGTCTGGAT-3ʹ |
| RBFox-2 | Fwd | 5ʹ-CCAACAAGAAGATGGTCACG-3ʹ |
|  | Rev | 5ʹ-TGTTGATGCCTCCTCTTCCT-3ʹ |
| RBFox-BS | Fwd | 5ʹ-GTAGTCGCATGGAGGAGAGC-3ʹ |
|  | Rev | 5ʹ-CTCAAGGATGGCTCAGCATAG-3ʹ |
| Claudin-1 | Fwd | 5ʹ-CTGGAAGATGATGAGGTGCAGAAGA-3ʹ |
|  | Rev | 5ʹ-CCACTAATGTCGCCAGACCTGAA-3ʹ |
| β-actin | Fwd | 5ʹ-GGCTACAGCTTCACCACCAC-3ʹ |
|  | Rev | 5ʹ-GAGTACTTGGCGTCAGGAGG-3ʹ |
| 36B4 | Fwd | 5ʹ-TGTGTGTCTGCAGATCGGGT-3ʹ |
|  | Rev | 5ʹ-TGGATCAGCCAGGAAGGCCT-3ʹ |

**Supplemental Table 3. 3ʹ-UTR sequences**

| RBFox-1 | Fwd | 5ʹ-GTTGTTTAAACGAGCTCACAGAGGGGCACACTTTGTGTGT -3ʹ |
| --- | --- | --- |
|  | Rev | 5ʹ- CGACTCTAGACTCGAGCAGATATGATTATCATGTAATTCC -3ʹ |
| TJAP-1 | Fwd | 5ʹ- GTTGTTTAAACGAGCTCGACCTGCCTGCCTTCTGCCACCAC -3ʹ |
|  | Rev | 5ʹ- CGACTCTAGACTCGAGGGGAGTAACAGTCTAGAGCCAAGGTT -3ʹ |
| Claudin-1 | Fwd | 5ʹ- GTTGTTTAAACGAGCTCCAGAGGCAAAGGAAGAGATCTTCCTGGAGC -3ʹ |
|  | Rev | 5ʹ- CGACTCTAGACTCGAGGTACAAATTCCCATTGCAGCCCCCAG -3ʹ |
