# Supplementary figures and images for: MiRNA-132/212 regulates tight junction stabilization in blood–brain barrier after stroke
Source: Cell Death Discov. 2021 Dec 8;7:380. doi: 10.1038/s41420-021-00773-w (PMC8654926; doi:10.1038/s41420-021-00773-w)

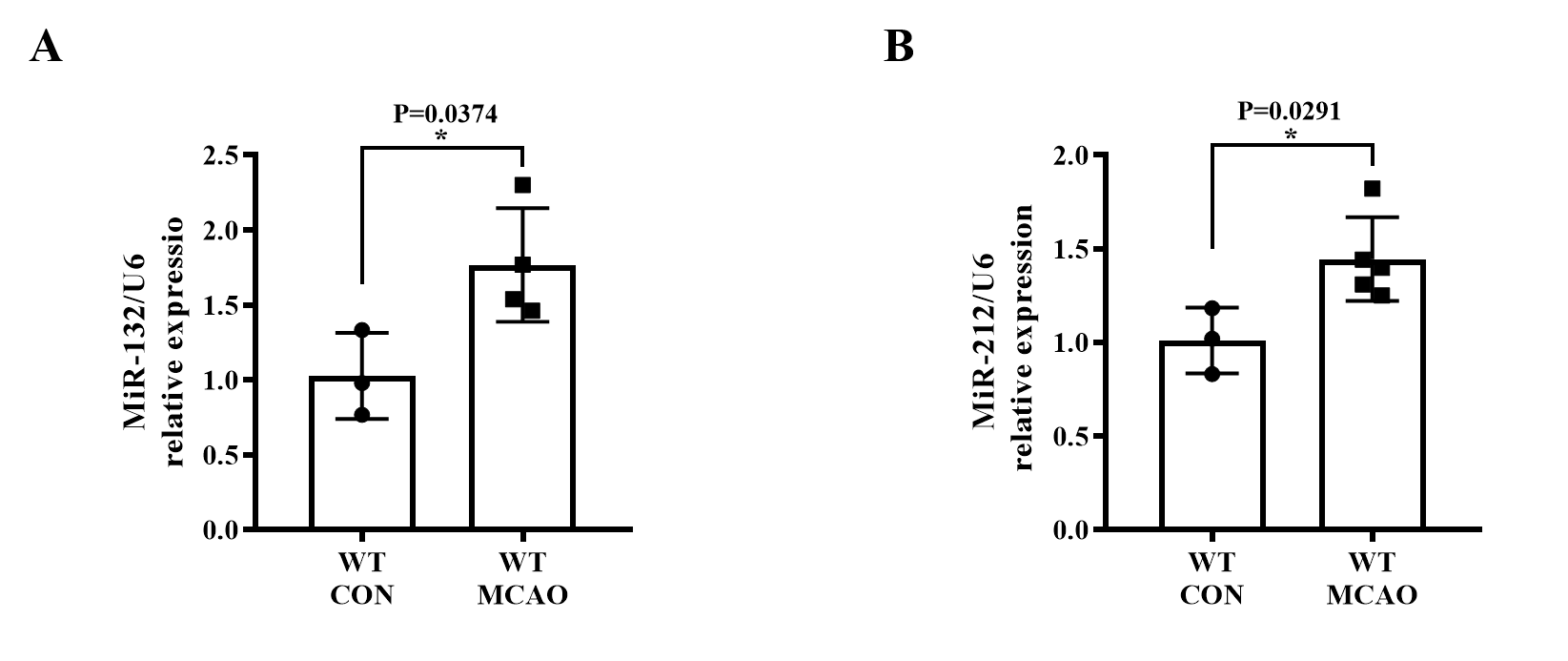

Supplement: Supplementary file 3 — Supplemental Figure 1 [file 41420_2021_773_MOESM3_ESM.tif]

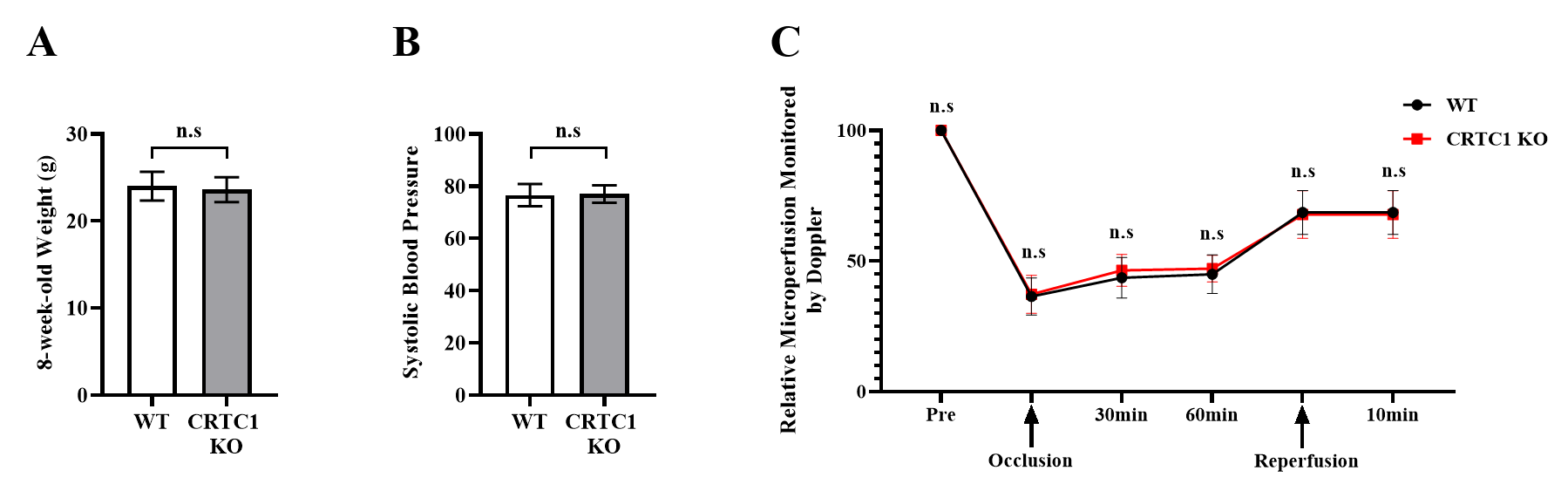

Supplement: Supplementary file 4 — Supplemental Figure 2 [file 41420_2021_773_MOESM4_ESM.tif]

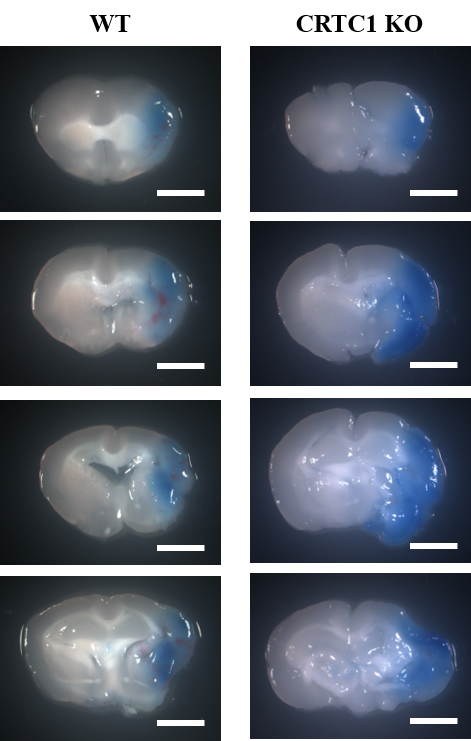

Supplement: Supplementary file 5 — Supplemental Figure 3 [file 41420_2021_773_MOESM5_ESM.tif]

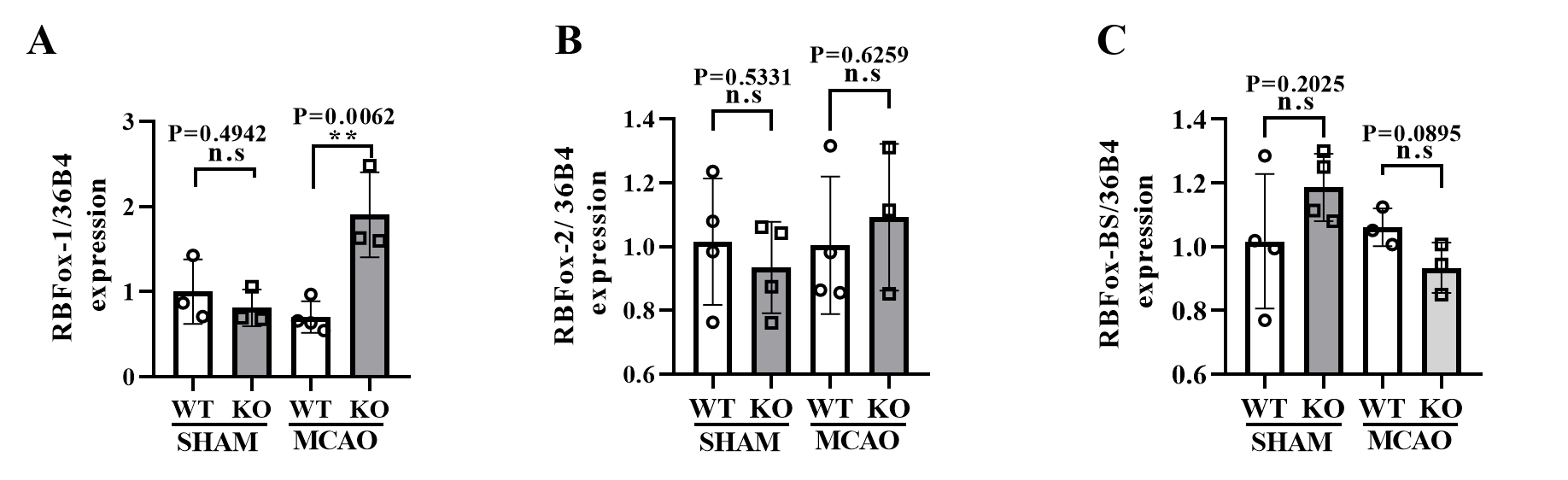

Supplement: Supplementary file 6 — Supplemental Figure 4 [file 41420_2021_773_MOESM6_ESM.tif]

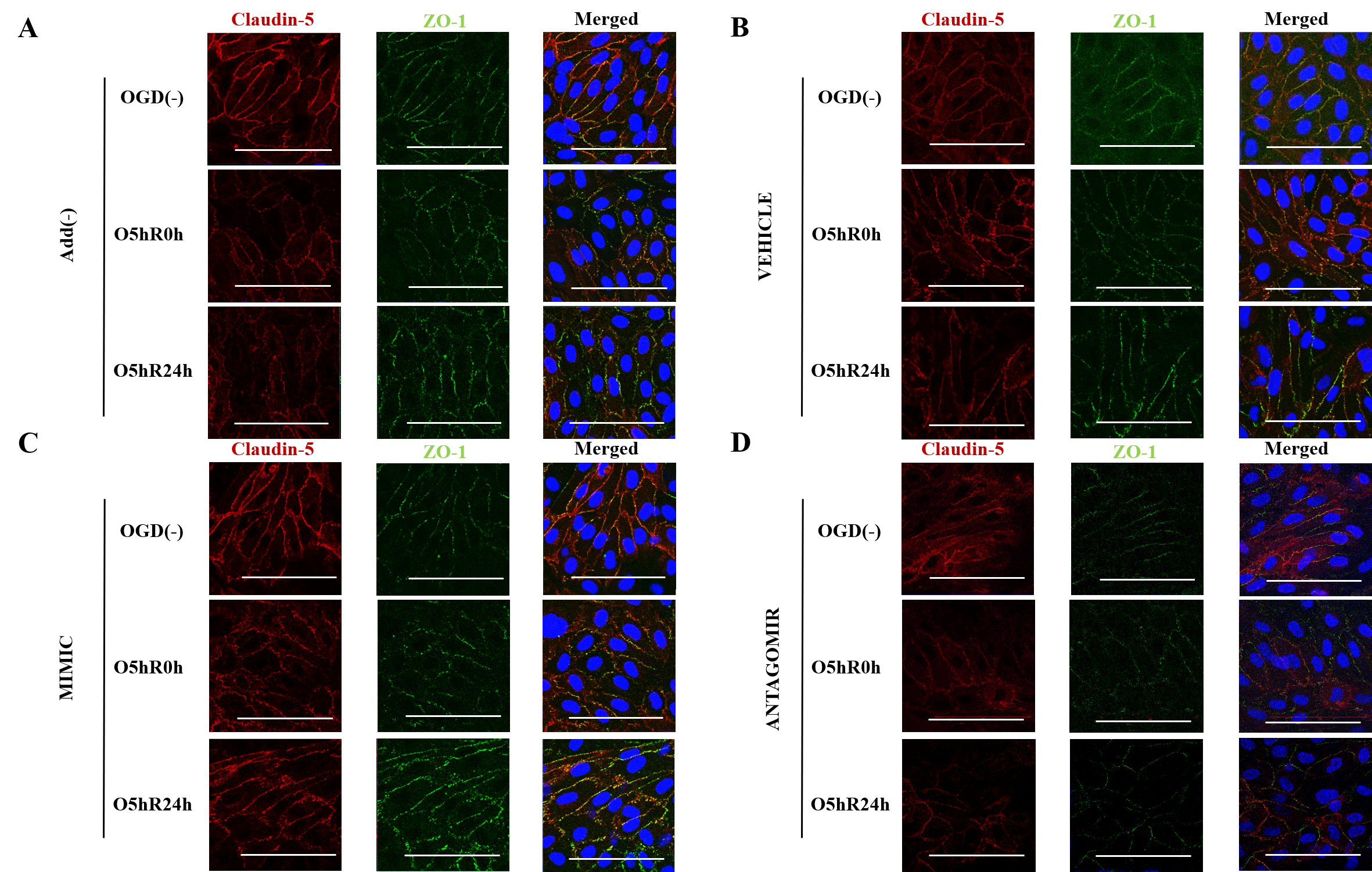

Supplement: Supplementary file 7 — Supplemental Figure 5 [file 41420_2021_773_MOESM7_ESM.tif]

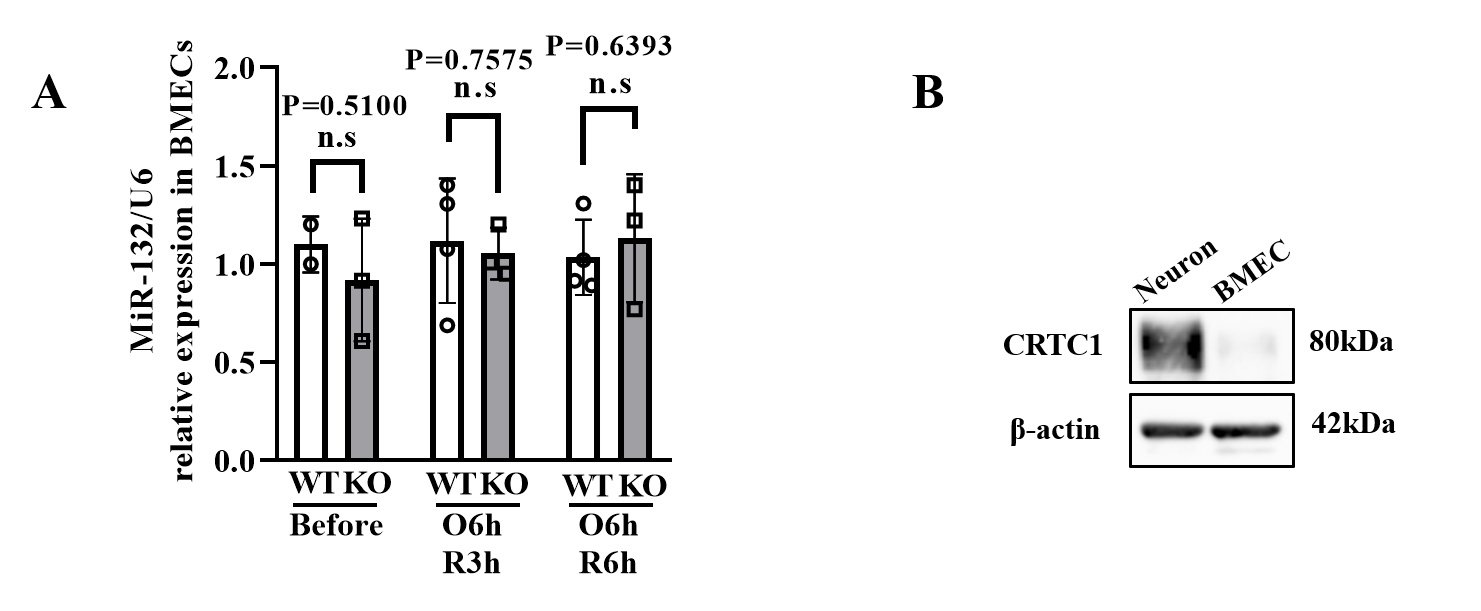

Supplement: Supplementary file 8 — Supplemental Figure 6 [file 41420_2021_773_MOESM8_ESM.tif]
