# Supplementary material for: MiRNA-132/212 regulates tight junction stabilization in blood–brain barrier after stroke
Source: Cell Death Discov. 2021 Dec 8;7:380. doi: 10.1038/s41420-021-00773-w (PMC8654926; doi:10.1038/s41420-021-00773-w)
Supplement: Supplementary file 9 — Supplemental Legends [file 41420_2021_773_MOESM9_ESM.docx]

**Supplementary Legends**

**Supplemental Table 1. Mortality rate of Mice**

**Supplemental Table 2. Primers sequences**

**Supplemental Table 3. 3ʹ-UTR sequences**

**Supplemental Figure 1. MiR-132/212 expression in penumbra of C57BJ/6 WT mice.** Transient MCAO induced a significant increase of miR-132 (**A**) and miR-212 (**B**) expression in mice penumbra. (n = 3 for SHAM, n = 5 for MCAO)

**Supplemental Figure 2. (A) 8-week-old weight and (B) systolic blood pressure of CRTC1 KO and WT mice. (C) Relative CBF change before and during operation of WT and CRTC1 KO mice, monitored by a laser-Doppler flowmetry attached to mice skull.**

**Supplemental Figure 3.** Representative Evans Blue images of CRTC1 KO and WT mice brain sections 24 hours after MCAO. (Interval, 2 mm. Scale Bar, 2 mm)

**Supplemental Figure 4. CRTC1 deletion did not alter RB-FOX2 and RB-FOXBS expression in mice, before and after MCAO.**

**Supplemental Figure 5.** Immunofluorescence of Claudin-5 and ZO-1 in HUVECs transfected with 50nM miR-132 mimic / antagomir / vehicle, subjected to OGD or not. (Scale Bar, 100um)

**Supplemental Figure 6. CRTC1 and miR-132 expression in BMECs.** CRTC1 deletion did not altered miR-132 expression in BMECs (**A**) and CRTC1 is specially expressed in neurons (**B**).
